# Fatty Acid Composition of Northern Pike from an Arctic River (Northeastern Siberia, Russia)

**DOI:** 10.3390/foods12040764

**Published:** 2023-02-09

**Authors:** Alexander G. Dvoretsky, Fatima A. Bichkaeva, Olga S. Vlasova, Sergei V. Andronov, Vladimir G. Dvoretsky

**Affiliations:** 1 Murmansk Marine Biological Institute of the Russian Academy of Sciences (MMBI RAS), 183010 Murmansk, Russia; 2 N. Laverov Federal Center for Integrated Arctic Research of the Ural Branch of the Russian Academy of Sciences (FECIAR UrB RAS), 163000 Arkhangelsk, Russia; 3 National Medical Research Center for Rehabilitation of Balneology, 121099 Moscow, Russia

**Keywords:** northern pike, fatty acids, northwestern Siberia, spatial variations, traditional diet

## Abstract

We assayed the fatty acid composition of muscles of the northern pike *Esox lucius* Linnaeus, 1758 inhabiting the Gyda River, Siberia, Russia using gas-liquid chromatography. Of 43 fatty acids identified in the pike samples, 23 fatty acids accounted for 99.3% of the total content. The most abundant saturated fatty acids (SFA, 31.6%) were palmitic (C16:0, 20.0%) and stearic (C18:0, 7.3%) acids. Among monounsaturated fatty acids (MUFA, 15.1%), oleic acid (C18:1n9, 10.2%) and palmitoleic acid (C16:1, 4.1%) demonstrated the highest levels. The most represented polyunsaturated fatty acids (PUFA, 53.3%) were arachidonic acid (C20:4n-6, 7.6%), eicosapentaenoic acid (EPA, C20:5n-3, 7.3%), and docosahexaenoic acid (DHA, C22:6n-3, 26.3%). The fatty acid profile of specimens from the Gyda River was different in comparison to profiles found in other pike populations, most likely due to different diets. Pike flesh has good nutrition quality in terms of a low n-6/n-3 ratio (0.36), low atherogenic (0.39), and thrombogenic (0.22) indices, and a high ratio of hypocholesterolemic to hypercholesterolemic fatty acids (2.83), and this species can be recommended as a replacement or alternative to other fish sources in traditional diets.

## 1. Introduction

In northwestern Siberia, remote Arctic communities have experienced relatively rapid shifts in lifestyle, including changes in diet [1]. In particular, consumption of a traditional subsistence diet has given way to an increased dependence on imported Western foods. This shift may play a significant role in a decline in diet quality and an increase in the occurrence of cardiovascular disease, obesity, and type 2 diabetes [2]. The diet of the indigenous inhabitants of Western Siberia’s Arctic zone such as the Khantys, Nenets, and Selkups is characterized by a major proportion of traditional food products. The traditional diet strongly depends on nomadic migrations and considers the seasonality of different fish species and fishing areas. The people of the Russian Arctic have traditionally used natural cryogenic resources such as snow, ice, and underground permafrost for short-term storing of fish and other products [1]. As the nutritional value and flavor characteristics of local fish products are significantly reduced during storage, fish is usually stored for a period which does not exceed three months.

The consumption of local products provides a person with a complete diet of fats, proteins, trace elements, vitamins, and biologically active substances as well as macro- and microelements [3]. The importance of the traditional diet for health and adaptation to the harsh conditions of the Arctic zone is not in doubt [4,5]. However, global climate change leads to dramatic changes in the hydrological regime of rivers including late freezing and early melting, which cause changes in the fishing calendar and the availability of natural storage reservoirs [6]. Decreasing consumption of traditional foods associated with a lower intake of essential fatty acids coupled with decreasing physical activity can result in increasing frequency of obesity and associated correlates, including atherosclerotic heart disease, diabetes, and dental caries [2]. Furthermore, the results of Andronov et al. [1] who studied the benefits of local products for indigenous peoples of northwestern Siberia found that the decreased duration of the consumption season and decreased consumption of local fish had adverse effects on health, leading to an increased rate of hypertension dissemination. Under such conditions, there is a growing demand for basic biological information regarding the chemical composition of different food products in order to choose alternatives to fish species whose stocks demonstrate negative trends. In recent decades, there has been a significant decrease in the abundance of some fish, including muksun, whitfish, and Arctic cisco, in the Russian Arctic [7], prompting a search for replacement alternatives to these fish without significant loss of quality. Potential candidates should have wide occurrence, high abundance, a relatively large size, and appropriate nutritional value. One fish species, the northern pike *Esox lucius*, which, in varying degrees, meets these criteria, has not been yet studied for its fatty acid composition in water bodies of the Gydan Peninsula. Here, this freshwater fish is a desirable object for fishing both by native inhabitants and anglers [8,9]. Northern pike have a circumpolar distribution in the Northern Hemisphere and the widest distribution of the living esocids [10]. As a cannibalistic top predator, the pike plays an important role in aquatic biotic communities [11]. This species is widely involved in recreational and commercial fisheries in the areas of their distribution [12]. Fishing methods include gillnets, fyke nets, trap nets, traps, long lines, hooks, and also active methods such as trawling, seining, angling, and spearing [13]. From 2015 to 2020, the global annual commercial capture rate of pike ranged from 27,500 to 32,123 t with the highest landings reported in Russia (60–65% of the total catch), Finland (18–27%), Canada (4–7%), Sweden (2–6%), and Uzbekistan (3–5%) [14]. Due to their voracity, cannibalistic behavior [15,16] and low survival rates even under favorable conditions with plenty of food [12], northern pike are considered unsuitable for farming [17], and pike aquaculture mainly focuses on producing fry and fingerlings for stocking natural waters [12].

Many aspects of the biology of northern pike are well described [18,19,20,21], and fatty acid profiles of *Esox lucius* from different habitats are also available in the literature [22,23,24,25,26,27,28].

The role of fatty acids in protection from many disorders and syndromes has been well demonstrated [29]. Consumption of fish and fish oil supplements rich in polyunsaturated fatty acids reduces all-cause mortality, cardiac and sudden death, and stroke [30]. Fatty acid profiles in fish may vary substantially depending on species, physiological status, season, and location [31,32].

Our aim was to study the fatty acid composition of northern pike from an Arctic population, calculate the health lipid indices of its flesh, and show the potential of this species to be a replacement alternative for traditional fish in the diet of local indigenous people who demonstrate a tendency to an increased obesity prevalence associated with the westernization of their diet. We also aimed to compare our data with fatty acid profiles established for other populations of northern pike.

## 2. Materials and Methods

### 2.1. Study Area and Sampling

Fish specimens were obtained from local authorized fishers just after catching in the Gyda River, located on the northern coast of the Gydan Peninsula near the Gyda settlement (70°53′41″ N, 78°30′14″ E) in September 2019. The local environmental regime is characterized by low-flux indoor lighting conditions [33], and as a result, a shorter period of ice-free water and lower water temperatures throughout the year. Deficiency of nutrients and low concentrations of dissolved oxygen are the main features of local water bodies, especially at the end of winter and in early spring [34]. The lack of oxygen is the main reason for fish kill events which affect many aspects of fish ecology at some sites [35]. In general, the water bodies of northwestern Siberia are classified as oligotrophic, with low diversity and productivity of fish communities.

The samples were taken from nine adult specimens (body length 47–49 cm, weight 3.2–3.4 kg). For biochemical assays, 0.8 g samples of white muscle tissue were taken in triplicate, 1–2 cm below the dorsal fin. The muscle samples were taken accurately to avoid undesirable components such as skin, red muscle, and bones. These samples were frozen and then transported to the laboratory of the Federal Center for Integrated Arctic Research (Arkhangelsk) for fatty acid analyses.

### 2.2. Biochemical Assay

Lipid fractions were first extracted from the pike samples following the Folch method [36], with slight modifications [37]. A detailed description is provided elsewhere [38,39,40]. Briefly, lipid samples were weighed and diluted in a chloroform–methanol mixture, with a solution of nonadecanoic acid used as the etherification reagent. As much as 3 mL of 0.74% of water solution of CaCl_2_ were added to a vial. The lower layer was used for further analysis after a 12 h storage period in a refrigerator. Afterward, 0.5–1.0 mL of methanol was added to the vial, and the sample was evaporated in a vacuum evaporator “Multivopar P12” (BÜCHI Labortechnik AG, Flawil, Switzerland). The extract was then dissolved in the chloroform–methanol mixture and shaken for 5 min. Two mL of 1.5% solution of H_2_SO_4_ in methanol was added, and the sample was then incubated in a water bath for 30 min at 90 °C and left for about 2–4 h until it separated into two phases. The top layer was pipetted, transferred to a 2-mL vial, and evaporated. Routine analysis of methyl esters was performed by a 7890 A gas chromatograph (Agilent Technologies Inc., Wilmington, DE, USA) equipped with a flame ionization detector and a capillary column Agilent DB-23 (60 m × 0.25 mm × 0.15 μm). The carrier gas was nitrogen, at a flow rate of 1 mL min^–1^. Each 200-μL sample was injected under the following programmed conditions. The oven temperature started at a constant temperature of 130 °C; it was then increased to 170 °C (rate 8.5 °C min^–1^), 206 °C (2 °C min^–1^), 220 °C (0.7 °C min^–1^), and 220 °C (6 °C min^–1^). The injector and detector temperatures were set to 270 °C and 280 °C, respectively. The resulting fatty acid methyl esters were identified using a standard mixture (qualitative and quantitative) with the known fatty acid components for verifications (Nu Chek Prep Inc 569 B) in Agilent Chem Station B.04.03 software.

Fatty acid concentrations (μg g^–1^ of sample) were used to calculate the atherogenic (*AI*), thrombogenic (*TI*), and *h/H* indices as follows [41]:

$$AI=\frac{C12:0+4\cdot C14:0+C16:0}{\sum MUFA+\sum n3+\sum n6}$$

$$TI=\frac{C14:0+C16:0+C18:0}{0.5\cdot \sum MUFA+0.5\cdot \sum n6+3\cdot \sum n3+\frac{\sum n3}{\sum n6}}$$

$$h/H=\frac{C18:1+C18:2+C18:3+C20:1+C22:1+C24:1}{C14:0+C16:0}$$

where ∑*MUFA* is the sum of monounsaturated fatty acids, *n*3 is omega-3 polyunsaturated fatty acids, and *n*6 is omega-6 polyunsaturated fatty acids.

### 2.3. Statistical Analysis

The mean value (*n* = 9) was calculated for each fatty acid and presented with standard error.

To reveal global spatial variations in fatty acid profiles of adult northern pike (%), we compared our data with other areas (Table 1).

Only the fatty acids that accounted for more than 0.4% of the total content were included in the comparative analysis.

Prior to multivariate analysis, the fatty acid data were square-root transformed. This transformation was used to better balance the weighting of both dominating and rare fatty acids on the similarity of samples. Similarities in the fatty acid composition between different locations were then calculated based on the Bray–Curtis similarity index. A Bray–Curtis similarity matrix with average linkage group classification was also used to produce a cluster dendrogram showing spatial variation in the fatty acid composition of northern pike. An analysis of similarity (ANOSIM) was used to detect significant differences between sampling sites with respect to fatty acid content. Recognition of fatty acids contributing to the separation between the spatial groups of northern pike delineated with the cluster analysis was carried out through the similarity percentages routine (SIMPER) in PRIMER 5.0.

## 3. Results

A total of 43 fatty acids were identified in northern pike samples, among which the proportion of >0.1% was registered for 23 fatty acids. Their sum accounted for 99.3% of the total content (Table 2).

The main saturated fatty acids (SFA) were palmitic (C16:0) and stearic (C18:0) acids. Their concentrations were found to be 812 ± 5 μg g^–1^ or 20.0 ± 0.3% and 296 ± 1 μg g^–1^ or 7.3 ± 0.1%, respectively. Among monounsaturated fatty acids (MUFA), oleic (C18:1n9c) and palmitoleic (C16:1n7c) demonstrated the highest levels: 416 ± 4 μg g^–1^ or 10.2 ± 0.2% and 166 ± 4 μg g^–1^ or 4.1 ± 0.1%, respectively. In northern pike, the most represented polyunsaturated fatty acid (PUFA) belonging to the n-6 PUFA family was arachidonic acid (C20:4n6), at 309 ± 6 μg g^–1^ or 7.6 ± 0.1%. Among n-3 PUFAs, the highest contents were found for docosahexaenoic acid (DHA, C22:6n3) and eicosapentaenoic acid (EPA, C20:5n3) averaging 1070 ± 34 μg g^–1^ or 26.3 ± 0.6% and 296 ± 5 μg g^–1^ or 7.3 ± 0.1%, respectively. PUFAs had the highest contribution to the total fatty acid content (55.3%) followed by SFAs (31.6%) and MUFAs (15.1%). As a result, the n-6/n-3 ratio was 0.36, while the PUFA/SFA ratio was 1.69. The atherogenic and thrombogenic indices were low (0.39 and 0.22, respectively), and the ratio between hypocholesterolemic and hypercholesterolemic fatty acids was 2.83.

**Figure 1:**
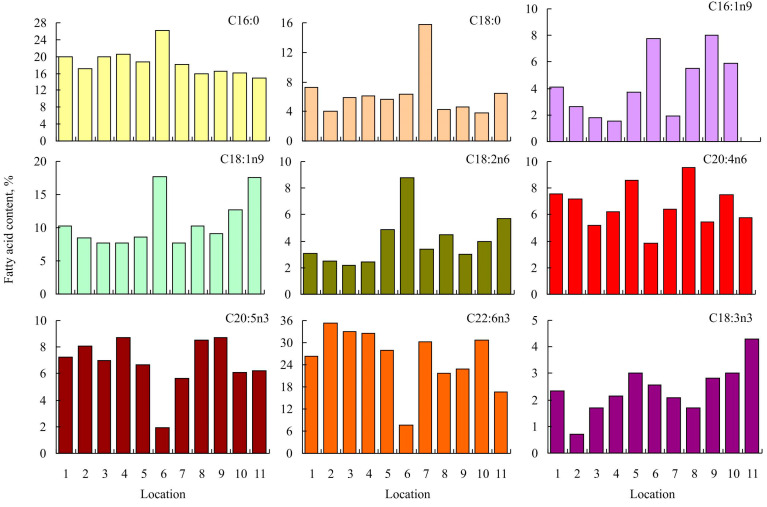
Spatial variations in the proportion of major fatty acids in the flesh of northern pike. Abbreviations: 1–Gyda River (Arctic, Russia), 2–Lake Sobachie (Arctic, Russia), 3–Ubei Bay (subarctic, Russia), 4–Lake Gusinoe (southern Siberia, Russia), 5–Lake Dgał Wielki (Poland), 6–Danube River (Serbia), 7–Lake Iseo (Italy), 8–Karamık Lake (Turkey), 9–Eber Lake (Turkey), 10–Cayuga Lake (USA), 11–Lac Ste. Anne (Canada).

**Figure 2:**
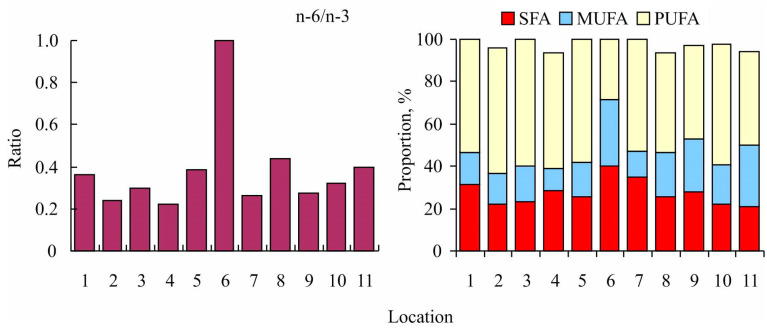
Spatial variations in the n-6/n-3 ratio and fatty acid contents in the flesh of northern pike. For abbreviations, see Figure 1 and Table 2.

In general, the percentages of major fatty acids in the muscles of pike from the Gyda River, as well as the n-6/n-3 ratio, were within the range established for this species in other locations (Figure 1 and Figure 2).

The predominance of PUFAs was consistent among different water bodies (44.2–59.3%) except for the Danube River, where the predominance of SFAs (40%) was recorded (Figure 2). Moreover, at the latter site, the n-6/n-3 ratio was 1, in contrast to other sites, at which this index varied from 0.22–0.44 (Figure 2).

**Figure 3:**
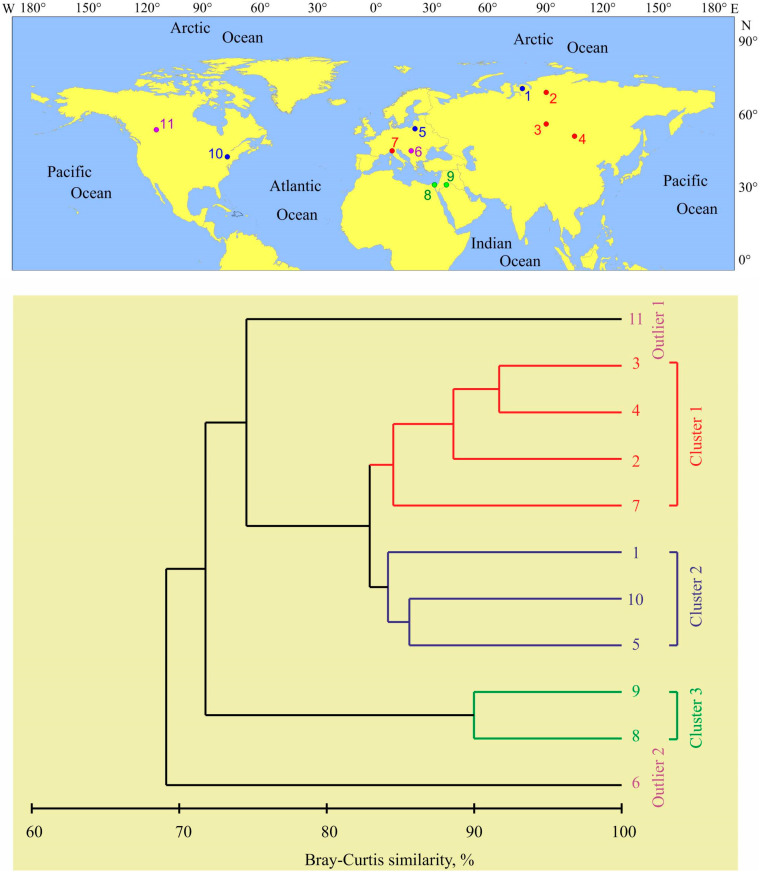
Hierarchical cluster dendrogram of the degree of similarity in the fatty acid content of pike from different locations. For abbreviations, see Figure 1.

Multivariate analysis based on square-root transformed fatty acid data yielded three distinct clusters at an 84% similarity level (Figure 3).

Group 1 consisted of four strongly related locations representing Lake Gusinoe, Ubei Bay, Lake Sobachie, and Lake Iseo (Figure 1). Group 2 included three sites (Gyda River, Dgał Wielki Lake, and Cayuga Lake), and Group 3 represented northern pike from Karamık Lake and Eber Lake (Figure 3). Two locations (Lac Ste. Anne and Danube River) were not grouped with any other cluster at a higher level of similarity, and were specified as outliers (Figure 1). There was a significant difference between the clusters (ANOSIM, Global R = 0.933, *p* < 0.001). Pair-wise comparisons showed significant differences in the total fatty acid content of individuals presented in Clusters 1 and 2 (R = 0.722, *p* = 0.029) and in Clusters 2 and 3 (R = 0.891, *p* = 0.048). There were no significant differences in other cases (R = 1.0, *p* = 0.1–0.3). SIMPER analysis identified fatty acids that characterized each cluster group (Table 3).

A total of six fatty acids (C16:1n9, C18:1n9, C16:1n7, C18:1n11, C22:5n6, and C22:6n3) contributed to most of the variations between the groups separated with cluster analysis. Dissimilarity levels and fatty acids with a high contribution to the dissimilarity revealed by the SIMPER for each pair of locations are presented in the Supplementary Material (Table S1). Specimens from the Gyda River demonstrated the highest degree of dissimilarity with pike inhabiting Lac Ste. Anne (Alberta), Danube River, Karamık Lake, and Eber Lake (Table S1). The fatty acid profiles of individuals collected in the Gyda River were the most similar to those revealed in pike from Lake Gusinoe, Lake Dgał Wielki, and Lake Cayuga (Figure 3, Table S1).

## 4. Discussion

Fish and seafood are considered the richest sources of n-3 PUFA [46]; however, as a result of overfishing, many natural populations have been demonstrated to be depleted. This has stimulated both aquaculture development and the expansion of fishing efforts to non-traditional and less explored fish species [47]. Our study is the first to report the fatty acid compositions of adult *Esox lucius* in the Gyda River. We found spatial variations in the fatty acid profiles of northern pike (Figure 1 and Figure 2). Spatio-temporal variability in fatty acid signatures of aquatic animals is a well-known pattern both in marine and aquatic environments [48,49,50]. Fatty acids are known to play a role in cell membrane fluidity, and the degree of saturation of phospholipid fatty acids directly correlates to water temperature [51]. Alterations in membrane fluidity following changes in the water temperature are known to result from adaptive changes of structural lipids which are associated with fluctuations in the content of unsaturated fatty acids accompanied by a reorganization of the molecular composition and architecture of phospholipids [52]. As a rule, cold-water species have a higher PUFA content than species from temperate habitats [48,49,53].

Previous studies have indicated that fatty acid compositions in fish are strongly affected by their trophic levels and food habits [54,55,56]. Northern pike are specialist piscivores that can grow to substantial size and have the capacity to consume prey of a large range of sizes. Across its range, pike consume predominantly cyprinid and percid fish, while at some sites, other fish families are more present in the diet of pike. In Turkey, Prussian carp *Carasius gibelio*, Menderes nase *Chondrostoma meandrense*, *Esox lucius*, western mosquitofish *Gambusia affinis*, gudgeon *Gobio gobio*, endemic cyprinid fish *Hemigrammocapoeta kemali*, common chub *Leuciscus cephalus*, and tench *Tinca tinca* are the most common food items by weight [57]. The presence of some unique fish in the diet may explain the grouping of two Turkish lakes into one cluster (Figure 3).

In Lake Cayuga, the major components of the diet of northern pike were smelt (*Osmerus mordax*), yellow perch (*Perca flavescens*), alewife (*Alosa pseudoharengus*), and whitefish (*Coregonus* sp.) [58]. In Poland, the adult specimens of pike fed on common roach (*Rutilus rutilus*), European perch *(Perca fluvatilis*), and ruffe (*Gymnocephalus cernuus*) [59]. In the Gyda River, the latter species together with smelt (*Osmerus mordax*) and juvenile *E. lucius* were important parts of the diet of northern pike. The presence of both smelt and perch results in close similarity among the fatty acid signatures of northern pike in the Gyda River, Lake Cayuga, and Lake Dgał Wielki (Figure 1, Figure 2 and Figure 3).

In Lake Iseo, the pike diet consisted mainly of invertebrates, terrestrial vertebrates, and fish [43]. This diet may explain the highest proportion of C18:0 in the total fatty acid content of pike in this area when compared to other sites (Figure 1). In Ubei Bay, the diet of pike mostly included fish (primarily roach), but crustaceans also contributed to the diet [27]. In Lake Sobachie (the Ob River Basin), the pike preyed on smaller conspecifics, perch, and cyprinids [35]. A similar, primarily fish-based diet is reported for pike in Lake Gusinoe [28]. Thus, it is not surprising that these Siberian sites are combined into one cluster (Figure 3).

According to Diana [60], the diet of adult pike in Lac Ste. Anne consisted predominantly of yellow perch (*Perca flavescens*), white sucker (*Catostomus commersoni*), burbot (*Lota lota*), and spottail shiner (*Notropis hudsonius*), which together comprised 94% of the total dietary caloric content. Most likely, the presence of these fish species explains the zero level of C16:1n9, the relatively high proportion of C18:1n9, and the low proportion of C22:6n3 in the total fatty acid content of northern pike in this lake (Figure 1). Another outlier is the Danube River. In this area, the dominating prey items in pike stomachs are crucian carp (*Carassius auratus gibelio*), common roach (*Rutilus rutilus*), and common bleak (*Alburnus alburnus*) [61]. Thus, a similar fatty acid content as at other European sites would be expected, but an extremely high SFA and extremely low PUFA content have been reported in this area [24]. The factors =responsible for this result are unknown, but we can assume that in the case of the Danube River, water pollution may at least partially explain the bias towards SFA in the total fatty acid content. The study site in the Danube River was located in Novi Sad [24], an industrial town in Serbia with sources of oil and heavy metal contamination [62,63]. Previous studies indicated that at contaminated sites, the SFA content in fish is higher than at non-polluted sites [64,65]. There are two main mechanisms explaining this result. First, pollutants lead to shifts in local food webs and therefore affect the diet of top predators [66]. Second, the accumulation of pollutants in fish tissues may cause alterations in fatty acid homeostasis and metabolism [67]. Further studies are required to reveal the mechanisms and processes responsible for spatial variations in the fatty acid profiles of northern pike as well as drivers of such variations in relation to food habits and environmental conditions. Fatty acid profiles in fish may vary substantially depending on sampling season, muscle type, and size (age) [7,31,68]. However, in our case, the impact of these factors seems to be not high enough, because all the authors considered adult fish, studied skeletal muscles, and sampled fish during the non-spawning seasons (Table 1) when the physiological status of northern pike is quite consistent.

There are clear species-specific variations in the fatty acid profiles of marine, freshwater, and anadromous fish, and the trophic position of a fish species, rather than sampling location or fish size, has been shown to be the best metric for explaining the proportion of total fatty acids made up by each fatty acid type [69,70,71]. In our study area, northern pike, a typical freshwater species, had lower total fatty acid concentrations than salmonid fish [7], i.e., predominantly anadromous species. The highest dereference in the total concentration (6.8 times) was found for the Arctic charr *Salvelinus alpinus,* with 39 cm length, and the lowest deference (2.8 times) was found for the least cisco *Coregonus sardinella,* with 15 cm length. On one hand, the proportion of PUFA in northern pike (53.3%) was higher than in salmonid fish, with the highest differences noted for Arctic charr and muksun *Coregonus muksun* (34.2 and 34.7%, respectively). At the same time, the proportion of MUFA (15.1%) in northern pike was 1.4–2.3 times lower than in salmonid fish [7].

Humans are inefficient in synthesizing long-chain PUFA and a certain amount of these essential substances need to be acquired directly from the diet [72]. The beneficial effects of long-chain n-3 PUFA on human health are well known, particularly the importance of having a ratio of n-6 and n-3 fatty acids biased toward n-3 PUFA. It has been established that 250 mg per day is the minimum quantity of EPA and DHA required for adults [73]. These compounds play a crucial role in reproduction processes, and for pregnancy and lactation, the adequate daily intake is set at 350–450 mg of DHA; meanwhile, for young children the suggested intake is 100 mg of DHA [74]. DHA is a key structural component of the membrane lipids of the human nervous system, and its adequate concentration is needed for optimal brain development [75]. In addition, n-3 PUFA consumption has beneficial effects in preventing Alzheimer’s and Parkinson’s disease [76] and reducing cardiovascular disease risks [77]. EPA and DHA are also considered important dietary components with anti-inflammatory and immunomodulating properties [78].

AI, TI, and *h/H* levels, as well as the PUFA n-6/n-3 ratios and PUFA/SFA, are commonly used indices to assess products’ nutritional value and the healthiness of intramuscular fat for human consumption. With regard to the PUFA/SFA and n-6/n-3 ratios, the recommended values are >0.45 and <4, while with respect to AI and TI, these parameters should not exceed 1 and 0.5, respectively. The higher the *h/H* index, the higher the nutritional value in terms of benefits for human health [41]. In our study area, the nutritional indices of northern pike all meet the requirements for healthy food. Moreover, as the flesh of northern pike contains less fat than the mentioned anadromous fish [7], its nutritional value is better, because it has a higher PUFA/SFA ratio (1.69 vs. 0.98–1.48), a higher *h/H* ratio (2.83 vs. 1.89–2.39) and a lower AI (0.39 vs. 0.50–0.68).

The caloric level of northern pike (402 Kcal) is lower than that of other Siberian fish such as muksun (492 Kcal), Arctic cisco *Coregonus autumnalis* (447 Kcal), least cisco (521 Kcal), and European whitefish *Coregonus lavaretus* (442 Kcal) [79]. Risk assessment models indicate that a daily consumption rate of the northern pike flesh accounts for 50 g; this level is lower than that established for muksun (440 g), broad whitefish *Coregonus nasus* (325 g), and least cisco (325 g) [80]. Currently, the consumption rate of northern pike is 74.4 g per day for indigenous people living in settlements and 20.4 g per day for indigenous people living in the tundra of northwestern Siberia [1]. The latter level is two times lower than recommended, suggesting that some actions in food policy are required to prevent arterial hypertension events, the prevalence of which has displayed a dramatic increase in the past decade in local ethnic communities [1]. Additional studies are required to reveal other chemical properties of this fish, including amino acid composition, flavor compounds, microelement composition, and sensory texture. Other fish species should also be studied for chemical composition to reveal the possibility of their use in the diet of indigenous peoples.

## 5. Conclusions

Fatty acid profiles of northern pike from the Gyda River were assayed for the first time. Our survey of the literature indicated spatial variations in the fatty acid profile of northern pike across the area of its distribution, most likely associated with differences in the pike diet. The muscle tissue of *Esox lucius* has good nutrition quality in terms of high content of essential fatty acids, an n-3/n-6 ratio close to optimal values, low indices of atherogenicity and thrombogenicity, and a relatively high *h/H* index. This fish is a good replacement for other fish in the traditional diet of indigenous peoples living in northwestern Siberia.

## Figures and Tables

**Table 1:** Information on water bodies inhabited by pike populations used for spatial comparisons of fatty acid profiles.

No.	Location	Country	Coordinates	Climatic Zone	Trophic Status	Period	Reference
1	Gyda River	Russia	70°53′41″ N, 78°30′14″ E	A	O	Sep	Present study
2	Lake Sobachie	Russia	69°02″ N 91°05″ E	A	O	Jul	[26]
3	Ubei Bay	Russia	55°55″ N 91°37″ E	SA	O	Oct	[27]
4	Lake Gusinoe	Russia	51°12′06″ N 106°23′32″ E	T	O	Jun	[28]
5	Lake Dgał Wielki	Poland	54°06′36″ N 21°47′35″ E	T	E	Aug	[42]
6	Danube River	Serbia	45°15′52″ N 19°49′54″ E	T	E	Nov	[24]
7	Lake Iseo	Italy	45°42′42″ N 10°04′19″ E	T	E	Jun	[43]
8	Lake Karamık	Turkey	32°28′ N 30°53′ E	W	E	Autumn	[44]
9	Lake Eber	Turkey	38°40′ N 31°12′ E	W	E	Aug	[23]
10	Lake Cayuga	USA	42°41′17″ N 76°42′8″ W	W	E	Sep/Oct	[45]
11	Lac Ste. Anne	Canada	53°44′ N 114°22 W	T	E	Jun/Aug	[22]

Note: A–Arctic, SA–subarctic, T–temperate, W–warm, O–oligotrophic, E–eutrophic.

**Table 2:** Fatty acid composition in the flesh of northern pike from the Gyda River.

Fatty Acid	Concentration, μg g^–1^				% of Total Fatty Acids			
	X	SE	Min	Max	X	SE	Min	Max
C6:0	0.35	0.01	0.32	0.38	0.01	0.00	0.01	0.01
C8:0	1.32	0.05	1.13	1.53	0.03	0.00	0.03	0.04
C9:0	1.19	0.02	1.12	1.27	0.03	0.00	0.03	0.03
C10:0	2.29	0.03	2.18	2.40	0.06	0.00	0.06	0.06
C11:0	0.80	0.01	0.76	0.85	0.02	0.00	0.02	0.02
C12:0	18.3	0.12	17.9	18.9	0.45	0.00	0.45	0.46
C13:0	25.0	0.04	24.9	25.2	0.62	0.00	0.60	0.63
C14:0	61.4	1.70	55.1	68.4	1.51	0.05	1.32	1.71
C15:0	30.3	0.50	28.4	32.3	0.75	0.02	0.68	0.81
C16:0	812	4.97	793	832	20.0	0.29	18.9	21.1
C17:0	25.2	0.16	24.6	25.8	0.62	0.01	0.58	0.65
C18:0	296	1.09	292	301	7.29	0.09	6.93	7.63
C20:0	3.72	0.01	3.68	3.76	0.09	0.00	0.09	0.10
C21:0	0.87	0.00	0.86	0.89	0.02	0.00	0.02	0.02
C22:0	3.07	0.02	3.01	3.14	0.08	0.00	0.07	0.08
C23:0	0.35	0.00	0.34	0.36	0.01	0.00	0.01	0.01
C24:0	2.72	0.06	2.52	2.95	0.07	0.00	0.06	0.07
C14:1n5t	0.28	0.01	0.23	0.33	0.01	0.00	0.01	0.01
C14:1n5c	0.95	0.06	0.71	1.21	0.02	0.00	0.02	0.03
C15:1	0.74	0.03	0.64	0.84	0.02	0.00	0.02	0.02
C16:1n7t	1.62	0.01	1.57	1.67	0.04	0.00	0.04	0.04
C16:1n7c	166	4.01	151	182	4.08	0.13	3.62	4.55
C17:1	3.02	0.05	2.85	3.21	0.07	0.00	0.07	0.08
C18:1n9t	6.58	0.21	5.81	7.42	0.16	0.00	0.15	0.18
C18:1n9c	416	3.63	402	431	10.2	0.17	9.59	10.9
C20:1	11.72	0.41	10.18	13.39	0.29	0.01	0.24	0.34
C22:1	1.02	0.01	1.00	1.04	0.03	0.00	0.02	0.03
C24:1	6.79	0.05	6.61	6.99	0.17	0.00	0.17	0.17
C18:2n6t	0.26	0.06	0.00	0.42	0.01	0.00	0.00	0.01
C18:2n6c	125	0.16	124	126	3.08	0.02	2.97	3.17
C18:3n3	94.7	0.04	94.6	94.9	2.33	0.02	2.24	2.41
C18:3n6	1.87	0.02	1.78	1.96	0.05	0.00	0.04	0.05
C20:2n6	18.7	0.04	18.5	18.9	0.46	0.01	0.44	0.48
C20:3n6	12.90	0.24	12.00	13.88	0.32	0.00	0.31	0.33
C20:4n6	309	5.95	287	333	7.59	0.08	7.30	7.88
C22:2n6	1.46	0.05	1.27	1.66	0.04	0.00	0.03	0.04
C20:5n3	295	4.99	277	316	7.25	0.06	7.04	7.46
C22:6n3	1070	33.50	947	1209	26.3	0.60	24.1	28.6
C20:3n3	9.94	0.01	9.92	9.97	0.24	0.00	0.23	0.25
C22:4n6	21.5	0.43	19.9	23.2	0.53	0.01	0.51	0.55
C22:3n3	0.43	0.01	0.41	0.46	0.01	0.00	0.01	0.01
C22:5n6	86.8	2.33	78.0	96.3	2.13	0.04	1.99	2.28
C22:5n3	119	2.91	108	131	2.92	0.05	2.75	3.09
∑SFA	1285	8.26	1254	1319	31.6	0.47	29.8	33.4
∑MUFA	614	7.87	585	647	15.1	0.32	14.0	16.2
∑PUFA	2170	50.6	1980	2376	53.3	0.78	50.4	56.1
Total	4069	35.8	3929	4232	100	0	100	100
n-3	1592	41.4	1437	1761	39.1	0.7	36.6	41.6
n-6	578	9.16	543	615	14.2	0.1	13.8	14.5
n-6/n-3	0.36	0.00	0.35	0.38	–	–	–	–
n-3/n-6	2.75	0.03	2.65	2.86	–	–	–	–
PUFA/SFA	1.69	0.05	1.51	1.88	–	–	–	–
*AI*	0.39	0.01	0.35	0.42	–	–	–	–
*TI*	0.22	0.01	0.19	0.24	–	–	–	–
*h/H*	2.83	0.07	2.57	3.10	–	–	–	–

Note. X–mean, SE–standard error, Min–minimum, Max–maximum, SFA–saturated fatty acids, MUFA–monounsaturated fatty acids, PUFA–polyunsaturated fatty acids, *AI*–atherogenic index, *TI*–thrombogenic index, *h/H*–ratio between hypocholesterolemic and hypercholesterolemic fatty acids.

**Table 3:** SIMPER (similarity percentages routine) results; fatty acids mostly contributing to the dissimilarity between clusters delineated based on square-root transformed concentrations of fatty acids in pike from different locations.

Pair	Dissimilarity, %	Fatty Acid (Contribution)
CL3–Out1	37.07	C16:1n7 (13%), C16:1n9 (10%), C24:1 (8%)
Out2–Out1	35.46	C16:1n7 (14%), C16:1n9 (12%), C18:1n11 (10%)
Out2–CL3	32.69	C18:1n11 (10%), C24:1 (8%), C22:6n3 (8%)
CL1–Out2	31.74	C22:6n3 (13%), C18:1n11 (10%), C8:0 (8%)
CL1–CL3	28.77	C24:1 (10%), C22:5n3 (8%), C16:1n9 (6%)
CL2–Out2	27.02	C22:6n3 (13%), C8:0 (9%), C18:1n11 (9%)
CL1–Out1	26.35	C16:1n7 (17%), C22:6n3 (10%), C16:1n9 (8%)
CL2–CL3	24.54	C24:1 (11%), C22:5n3 (10%), C22:5n6 (8%)
CL2–Out1	24.25	C16:1n7 (21%), C16:1n9 (13%), C22:6n3 (8%)
CL2–CL1	17.09	C18:1n7 (10%), C16:1n9 (6%), C18:0 (5%)

Note. For cluster groups, see Figure 1.

## Data Availability

The data presented in this study are available on request from the corresponding author.

## Supplementary Materials

The following supporting information can be downloaded at: https://www.mdpi.com/article/10.3390/foods12040764/s1, Table S1. Results of similarity percentage (SIMPER) comparison between pike inhabiting different locations, to find fatty acids that best distinguish between the sites.
