# Supplementary material for: Fatty Acid Composition of Northern Pike from an Arctic River (Northeastern Siberia, Russia)
Source: Foods. 2023 Feb 9;12(4):764. doi: 10.3390/foods12040764 (PMC9955493; doi:10.3390/foods12040764)
Supplement: Supplementary file 1 [file foods-12-00764-s001.zip › foods-2192640-supplementary.pdf]

Supplementary Material

Table S1. Results of similarity percentage (SIMPER) comparison between pike inhabiting different locations, to find fatty acids that best distinguish between the sites.

Pair	Dissimilarity, %	Fatty Acids (Contribution)
Ar-GR-Rus – Ar-LS-Rus	16.64	C18:1n7 (14%), C22:6n3 (7%), C13:0 (6%)
Ar-GR-Rus – Sub-UB-Rus	18.39	C18:1n7 (12%), C18:4п-3 (7%), C13:0 (6%)
Ar-GR-Rus – Temp-DR-Ser	25.49	C22:6n3 (12%), C18:1n11 (12%), C8:0 (9%)
Ar-GR-Rus – Temp-DWL-Pol	15.36	C18:1n11 (14%), C18:4п-3 (7%), C17:0 (7%)
Ar-GR-Rus – Temp-GL-Rus	16.11	C16:1n7 (9%), C13:0 (7%), C16:1n9 (7%)
Ar-GR-Rus – Temp-IL-It	19.68	C18:1n7 (11%), C22:5n6 (10%), C18:0 (9%)
Ar-GR-Rus – Temp-LS-Can	28.32	C16:1n7 (17%), C16:1n9 (10%), C14:0 (6%)
Ar-GR-Rus – Warm-CL-USA	16.34	C15:0 (7%), C17:0 (7%), C13:0 (7%)
Ar-GR-Rus – Warm-EL-Tur	20.5	C24:1 (12%), C22:5n3 (11%), C22:5n6 (9%)
Ar-GR-Rus – Warm-KL-Tur	20.5	C22:5n3 (11%), C22:5n6 (9%), C24:1 (9%)
Ar-LS-Rus – Temp-DR-Ser	32.52	C22:6n3 (14%), C18:1n11 (10%), C8:0 (8%)
Ar-LS-Rus – Temp-IL-It	13.93	C18:0 (20%), C22:5n6 (10%), C18:3n3 (6%)
Ar-LS-Rus – Temp-LS-Can	28.26	C16:1n7 (16%), C22:6n3 (10%), C18:1n7 (9%)
Ar-LS-Rus – Warm-CL-USA	17.08	C18:1n7 (15%), C18:3n3 (8%), C14:0 (7%)
Ar-LS-Rus – Warm-EL-Tur	27.43	C24:1 (11%), C22:5n3 (9%), C18:1n7 (6%)
Ar-LS-Rus – Warm-KL-Tur	27.14	C24:1 (10%), C22:5n3 (9%), C18:1n7 (9%)
Sub-UB-Rus – Ar-LS-Rus	10.38	C20:2n6 (9%), C20:3n6 (8%), C18:3n3 (7%)
Sub-UB-Rus – Temp-DR-Ser	32.61	C22:6n3 (13%), C18:1n11 (10%), C8:0 (8%)
Sub-UB-Rus – Temp-IL-It	14.75	C18:0 (15%), C22:5n6 (14%), C22:4n6 (9%)
Sub-UB-Rus – Temp-LS-Can	23.86	C16:1n7 (18%), C22:6n3 (11%), C18:1n7 (10%)
Sub-UB-Rus – Warm-CL-USA	17.88	C18:1n7 (13%), C16:1n9 (9%), C22:4n6 (9%)
Sub-UB-Rus – Warm-EL-Tur	29.3	C24:1 (11%), C16:1n9 (7%), C22:5n3 (7%)
Sub-UB-Rus – Warm-KL-Tur	30.1	C24:1 (9%), C18:1n7 (7%), C22:5n3 (7%)
Temp-DR-Ser – Temp-LS-Can	35.46	C16:1n7 (14%), C16:1n9 (12%), C18:1n11 (10%)
Temp-DR-Ser – Warm-CL-USA	30.28	C22:6n3 (13%), C18:1n11 (11%), C8:0 (9%)
Temp-DR-Ser – Warm-EL-Tur	32.87	C18:1n11 (9%), C24:1 (9%), C22:6n3 (8%)
Temp-DR-Ser – Warm-KL-Tur	32.5	C18:1n11 (10%), C8:0 (8%), C22:6n3 (8%)
Temp-DWL-Pol – Ar-LS-Rus	16.74	C18:1n7 (15%), C18:1n11 (14%), C18:3n3 (8%)
Temp-DWL-Pol – Sub-UB-Rus	14.56	C18:1n11 (16%), C18:1n7 (15%), C20:2n6 (8%)
Temp-DWL-Pol – Temp-DR-Ser	25.28	C22:6n3 (14%), C8:0 (10%), C22:5n6 (8%)
Temp-DWL-Pol – Temp-IL-It	17.7	C18:1n11 (13%), C18:0 (13%), C18:1n7 (12%)
Temp-DWL-Pol – Temp-LS-Can	24.13	C16:1n7 (21%), C16:1n9 (12%), C18:1n11 (10%)
Temp-DWL-Pol – Warm-CL-USA	14.4	C18:1n11 (17%), C22:4n6 (11%), C20:2n6 (8%)
Temp-DWL-Pol – Warm-EL-Tur	27.56	C24:1 (11%), C22:5n3 (8%), C18:1n11 (8%)
Temp-DWL-Pol – Warm-KL-Tur	25.17	C24:1 (10%), C22:5n3 (9%), C18:1n11 (9%)
Temp-GL-Rus – Ar-LS-Rus	12.44	C18:1n7 (21%), C22:5n3 (10%), C18:3n3 (8%)
Temp-GL-Rus – Sub-UB-Rus	8.31	C18:1n7 (28%), C22:5n3 (9%), C22:5n6 (8%)
Temp-GL-Rus – Temp-DR-Ser	30.18	C22:6n3 (14%), C18:1n11 (11%), C8:0 (9%)
Temp-GL-Rus – Temp-DWL-Pol	15.1	C18:1n11 (16%), C16:1n7 (10%), C22:5n3 (7%)
Temp-GL-Rus – Temp-IL-It	17.79	C18:1n7 (13%), C18:0 (12%), C22:5n3 (10%)
Temp-GL-Rus – Temp-LS-Can	23.33	C16:1n7 (16%), C22:6n3 (11%), C18:1n9 (10%)
Temp-GL-Rus – Warm-CL-USA	17.76	C16:1n9 (10%), C22:4n6 (9%), C16:1n7 (9%)
Temp-GL-Rus – Warm-EL-Tur	26.83	C24:1 (12%), C16:1n9 (8%), C24:0 (6%)

Temp-GL-Rus – Warm-KL-Tur	26.78	C24:1 (10%), C16:1n9 (6%), C24:0 (6%)
Temp-IL-It – Temp-DR-Ser	31.65	C22:6n3 (12%), C18:1n11 (10%), C8:0 (8%)
Temp-IL-It – Temp-LS-Can	29.94	C16:1n7 (17%), C18:1n7 (8%), C18:0 (7%)
Temp-IL-It – Warm-CL-USA	17.41	C18:0 (17%), C18:1n7 (13%), C22:5n6 (10%)
Temp-IL-It – Warm-EL-Tur	30.02	C24:1 (10%), C22:5n3 (10%), C18:0 (8%)
Temp-IL-It – Warm-KL-Tur	32.51	C22:5n3 (9%), C18:0 (8%), C24:1 (8%)
Warm-CL – USA-Temp-LS-Can	20.28	C16:1n7 (27%), C16:1n9 (19%), C22:6n3 (12%)
Warm-EL-Tur – Temp-LS-Can	38.22	C16:1n7 (13%), C16:1n9 (11%), C24:1 (9%)
Warm-EL-Tur – Warm-CL-USA	25.93	C24:1 (12%), C22:5n3 (10%), C15:0 (7%)
Warm-KL-Tur – Temp-LS-Can	35.92	C16:1n7 (14%), C16:1n9 (10%), C24:1 (8%)
Warm-KL-Tur – Warm-CL-USA	27.57	C22:5n3 (10%), C24:1 (10%), C15:0 (7%)
Warm-KL-Tur – Warm-EL-Tur	10.01	C22:4n6 (11%), C20:4n6 (10%), C14:0 (9%)

Abbreviations: Ar-GR-Rus – Gyda River (Arctic, Russia), Temp-GL-Rus – Lake Gusinoe (Russia), Temp-DWL-Pol – Dgał Wielki Lake (Poland, Europe), Sub-UB-Rus – Ubei Bay (sub-Arctic, Russia), Ar-LS-Rus – Sobachie Lake (Arctic, Russia), Temp-IL-It – Iseo Lake (Italy, Europe), Temp-DR-Ser – Danube River (Serbia, Europe), Warm-KL-Tur – Karamık Lake (Turkey), Warm-EL-Tur – Eber Lake (Turkey), Warm-CL-USA – Cayuga Lake (USA), Temp-LS-Can – Lac Ste. Anne (Canada).
